# Who should be tested in a pandemic? Ethical considerations

**DOI:** 10.1186/s12910-021-00640-4

**Published:** 2021-06-22

**Authors:** Sven Ove Hansson, Gert Helgesson, Niklas Juth

**Affiliations:** 1 Department of Learning, Informatics, Management and Ethics, Karolinska Institutet, 171 77 Stockholm, Sweden; 2 Division of Philosophy, KTH Royal Institute of Technology, Teknikringen 76, 100 44 Stockholm, Sweden

**Keywords:** Covid-19, Pandemic, Testing, Priority-setting, Direct-to-consumer tests, Unauthorized immigrants, Obligatory testing

## Abstract

**Background:**

In the initial phase of the Covid-19 pandemic, difficult decisions had to be made on the allocation of testing resources. Similar situations can arise in future pandemics. Therefore, careful consideration of who should be tested is an important part of pandemic preparedness. We focus on four ethical aspects of that problem: how to prioritize scarce testing resources, the regulation of commercial direct-to-consumer test services, testing of unauthorized immigrants, and obligatory testing.

**Main text:**

*The distribution of scarce resources for testing*: We emphasize the use of needs-based criteria, but also acknowledge the importance of choosing a testing strategy that contributes efficiently to stopping the overall spread of the disease. *Commercial direct-to-consumer test services*: Except in cases of acute scarcity, such services will in practice have to be allowed. We propose that they should be subject to regulation that ensures test quality and adequate information to users. *Testing of unauthorized immigrants, their children and other people with unclear legal status*: Like everyone else, these individuals may be in need of testing, and it is in society’s interest to reach them with testing in order to stop the spread of the disease. A society that offers comprehensive medical services to unauthorized immigrants is in a much better position to reach them in a pandemic than a society that previously excluded them from healthcare. *Obligatory testing*: While there are often strong reasons for universal testing in residential areas or on workplaces, there are in most cases better ways to achieve testing coverage than to make testing mandatory.

**Conclusion:**

In summary, we propose (1) decision-making primarily based on needs-based criteria, (2) strict regulation but not prohibition of direct-to-consumer test services, (3) test services offered to unauthorized immigrants, preferably as part of comprehensive medical services, and (4) broad outreach of testing services whenever possible, but in general not obligatory testing.

*“[T]he ethical issues raised by infectious diseases are often related to these diseases’ powerful ability to engender fear in individuals and panic in populations. This fear and panic often leads to rapid, emotionally driven decision making about the care of individual patients and about public health policies, even when these decisions challenge generally accepted medical-ethics principles such as patient autonomy, non-maleficence, beneficence and justice.”* [57], p. 3

## Introduction

One of the many contentious issues in the Covid-19 pandemic has been the use of, and access to, tests for the disease. How can testing best be used as part of a strategy to curb and conquer the pandemic, and how should such testing be organized? Much can be learned from previous discussions on screening and other preventive measures, but the new situation of a rapidly spreading pandemic requires new deliberations. The present contribution is devoted to the most basic question in this context, namely: who should be tested? This involves both medical and ethical considerations; our focus is on the latter. We will deal primarily with issues that have arisen or are expected to arise in the current Covid-19 pandemic, but much of this will also be relevant in coming pandemics and large epidemics.

Next we provide a brief background on the currently available tests. Our discussions of the ethical issues are divided into four sections dealing with various practices that determine who will be tested, namely (1) priority-setting when resources for testing are scarce, (2) direct-to-consumer sale of tests, (3) testing of people with unclear legal status, and (4) whether testing should be mandatory.

## Background

Two main types of tests for Covid-19 are available. [40] Virus tests detect the presence of the virus, which indicates an active infection. Antibody tests detect the presence of antibodies against the virus. In persons not currently infected by the virus, a positive antibody test indicates a previous infection and is taken as indicative of immunity.

*Virus testing* is usually performed on nasopharyngeal or oropharyngeal specimens, collected with a swab and analysed with RT-PCR (real-time polymerase chain reaction) technology. The test answers a yes-or-no question, namely whether the person is (at all) currently infected by the virus. Therefore, the criteria for sensitivity and specificity of such tests are well defined. The major problem with interpreting a virus test is to make sure that persons who test negative are informed of the short duration of the conclusion that they do not carry the disease. Unless this is fully understood and observed, negative test outcomes can potentially encourage risky behaviour. It should also be observed that inaccurate specimen collection can result in false negative tests, which suggests that swabbing should preferably be performed by professionals.

*Antibody tests* are blood tests performed to determine the level of antibodies against the virus in the individual’s serum. This is a quantitative, rather than a yes-or-no question, and it is assumed that more antibodies are associated with better chances of immunity. At the time of writing, the strength of that presumed correlation is unknown. It is also unclear to what extent other mechanisms than antibody formation contribute to immunity against SARS-CoV-2. Likewise, the duration of acquired immunity against the virus is unknown.

For tests that measure a variable coming in degrees, sensitivity and specificity are not unequivocally defined. To define them, a threshold value has to be decided, and sensitivity and specificity will have to be related to that value. In this case, the threshold is an amount of antibodies present in the serum. If the test is intended for individual use, then positive test outcomes will expectedly lead to reduced caution against transmission of the disease. This means that the tolerance for false positives will have to be very low.

Antibody tests can also be used for population studies of the proliferation of the disease and the development of population-level immunity. For that purpose, tests with a lower specificity (higher rate of false positives) could in principle be used. In practice, however, it is unlikely that the individual results of such testing can be kept a secret from those tested. A policy of secrecy would also violate the right to know, firmly based in considerations of both autonomy and privacy [30]. Hence, most of those whose antibody levels are measured in a population study should and likely will get to know their results. This will in practice make the avoidance of false positives equally important in population studies as in tests offered for individual use. The statistical analysis on population level need not be restricted to the dichotomy between positive and negative outcomes, but can also make use of the exact antibody titres.

In the following, we will focus on the use of virus and antibody tests to guide diagnosis and acute containment measures. In the clinic, a variety of other tests are needed, for instance to detect the cytokines storm that occurs in a subgroup of patients and may require specific (immunosuppressive) therapy. Tests are also needed in vaccine evaluation, and the evolution of the virus has to be carefully followed with RNA sequencing. Such tests will not be discussed here.

## Priority-setting

In an epidemic with a previously unknown pathogen, no vaccine is initially available. If vaccine development is successful, this can be followed by a phase in which vaccine is available but in insufficient quantity. Access to vaccine will then have to be prioritized [64]. Experience from Covid-19 has shown that the situation is similar for tests, although their development is faster than for vaccines. (This largely due to the trial phases in vaccine development, which were completed in unprecedentedly short time for Covid-19 [18]). Although virus tests were launched early in the pandemic, the initial tests required laboratory analysis. In combination with logistic hurdles and lack of equipment, this led to a shortage in the capacity for mass testing, even in industrialized countries. Testing capacity has been substantially increased through reassignment and reorganization of laboratory resources. It has been further increased by the development of rapid diagnostic tests (RDT), which do not require access to a laboratory. These tests are suitable both for point-of-care testing and screening. However, rapid tests are often of a lower quality, and they generate more false answers than laboratory testing. We are therefore facing two priority questions: who should be prioritized when it comes to testing and who should be prioritized when it comes to high-quality testing? Both these questions have much in common with the prioritization of scarce vaccines. However, there are also differences. For instance, contrary to vaccination, testing can potentially give rise to restrictive measures such as quarantining. The priority-setting of testing and vaccination should therefore be treated as different issues.

Experience from other diseases such as malaria shows that access to RDTs is essential in countries with less developed laboratory resources [29]. The diagnostic situation for Covid-19 would have been much worse if the initial spread had taken place in countries with less developed laboratory resources. Therefore, capacity for speedy development and mass production of RDTs should be recognized as an essential component of global preparedness for epidemics with new infectious agents.

Lack or scarcity of testing resources is unavoidable in the early phases of a new infectious disease. In addition, scarcity can remain due to logistic inefficiency and/or lack of resources. This gives rise to a triage situation for the distribution of available testing capacity. The standard approach to prioritization in publicly funded health care is to base it on health care needs or potential benefits. (Sometimes principles of need are even understood partly in terms of potential benefits.) [24]. Needs, in turn, are typically understood in terms of severity: the worse off you will be unless the health care intervention is provided, the greater the need. This approach favours the assignment of testing capacities primarily to testing for the virus rather than for antibodies, since the former type of test provides information that is essential for the distribution of healthcare resources. Furthermore, this line of reasoning indicates that tests should be offered to those who would stand to lose the most if not tested. This is both intuitively appealing and caters consideration of justice and fairness [31].

In the current pandemic, individuals need testing primarily in order to obtain timely and adequate treatment. The need for early diagnosis in order to receive early treatment is largest for those who run a high risk of a severe development of the disease. This is a strong reason to prioritize virus testing of groups that are more likely to die or suffer seriously from Covid-19. Studies of previous pandemics show that mortality has usually been highest in the population groups with the lowest socio-economic status [42]. The socioeconomic profile of Covid-19 mortality has a similar pattern [34, 65]. High age and certain comorbidities substantially increase the mortality of Covid-19 patients [46, 51]. Needs-based considerations support the prioritization of these risk groups for testing.

However, even if a decision is made to base priority setting on medical needs, this does not settle all issues in priority setting. Important interpretational issues remain. A high probability of death and severe symptoms increases a person’s need of testing, but arguably so do a large potential loss of life years and a substantial reduction of health-related quality of life. These factors sometimes pull in opposite directions. Take for instance age: the older you are, the more likely it is that you die of Covid-19 or suffer serious symptoms. However, on the other hand, with older age follows a lower number of expected life years that can be lost. This will strengthen the justice- or needs-based claims of younger people to be prioritized. In the end, this argument requires that we balance the probability of mortality against the expected number of life years that would be lost in the case of a fatal outcome. For Covid-19 it has been shown that vaccinating the oldest first not only saves the largest number of lives, but also maximizes the total number of remaining life years [23]. It is a plausible assumption that the gains obtained by testing have a similar distribution. For another pandemic disease with a different mortality pattern, this may be different. At any rate, the described value conflict needs to be recognized.

Moreover, there are other considerations than only justice- or needs-based ones. There is an essential public health dimension in large-scale testing for Covid-19 and other pandemic diseases. Testing is essential for the targeting of preventive measures and for planning the medical response to the pandemic. Hence, what matters is not only a just distribution of testing, but also the dynamic effects of testing, i.e. the overall outcome. Testing that would not contribute much to curbing the pandemic may not be justified if the resources can be used for other, more efficient measures against the disease. For instance, at some stages of the pandemic it may be even more important to identify infected individuals with the greatest risk of spreading the disease than to identify those in greatest need of treatment. It may also be harder to justify large-scale population screening for the virus than testing that traces the spread of the virus in local outbursts. In the latter case, costs are limited and chances are usually good that the test outcomes can be used efficiently to contain the disease. Population screening may be useful if there is a lack of reliable information on the social spread of the disease, but if such information is already available, then the new information obtainable by (additional) screening may not be worth the resources it requires. Considerations like these can lead to a strategy that is adjusted to maximize the overall impact on the spread of the pandemic. In such a strategy, considerations concerning the just distribution of opportunities to be tested will be relegated to a secondary role.

In this broader public health perspective there is a special need for virus testing of health care personnel that care for infected patients. They are a scarce resource, and life-saving healthcare is threatened if the disease spreads among them. Furthermore, access to testing can be seen as a partial compensation for their exposure to risk [47].

Public health goals are more complicated than what first meets the eye. Often, it is presupposed that minimizing Covid-19 mortality is the most important goal. However, there are reasons to also consider other, more over-arching health goals. It is not clear that lower rates of premature death are best achieved by minimizing the mortality directly caused by the pandemic virus. Unfortunately, in particular in poor countries, some of the measures taken to fight Covid-19 tend to have unintended (although often foreseen) negative health effects. The disruption of routine vaccination has put at least 80 million children at risk of potentially deadly diseases such as diphtheria, polio and measles.^1^ Increased poverty and lack of public transportation due to Covid-19 lockdowns have prevented, and continue to prevent, people’s access to healthcare. According to one estimate, six months of impeded maternal and child care due to pandemic lockdowns will result in at least 250,000 additional child deaths and 12,000 maternal deaths [52]. Another serious concern is the increased prevalence of child malnutrition that follows from the disruption of food systems in low- and middle-income countries [28]. There are also indications that, even in rich countries, decreased use of emergency care for life-threatening diseases unrelated to Covid-19 may have led to excess mortality [37]. In particular in poor countries with severely limited healthcare resources, the prioritization of testing has to be considered not only in relation to other possible measures against the pandemic, but also in relation to other measures that are important for public health.

In summary, a country’s priority-setting for testing will have to depend on several factors such as the dissemination of the disease, the patterns of disease transmission, the country’s access to healthcare resources, and its situation with respect to the provision of food, water, and other basic conditions for human life.

The distribution of tests is not only a national but also to a high degree an international issue. The situation is analogous with that for vaccine distribution. Unfortunately, the initial distribution of Covid-19 vaccines among nations is largely based on their ability to pay, leading to a scarcity in poor countries that cannot be justified from a medical or humanitarian point of view. An interesting proposal has been made that vaccines should be distributed between countries according to a principle of “fair priority”. In a first phase with extreme shortage of vaccine, this principle implies that the distribution of vaccine between countries should aim at maximizing the expected number of life-years saved [16]. The same basic principles can be applied to scarce testing resources, for instance RDT equipment, although the details of such a distribution remain to develop.

## A market for testing those who can pay for it?

In the Covid-19 pandemic there has been a considerable demand from the public for both virus tests and antibody tests, often for purposes that would not be prioritized according to the approaches to priority setting discussed above. Symptom-free persons may want a virus test to confirm that they do not have the infection, or an antibody test for immunity assurance. Many countries have plans for the introduction of “Covid-19 immunity passports” that would allow people to work and travel. [8, 35]

As indicated in above, there are considerable problems with such uses of the tests. The virus test (PCR test) had problems with false negative results in an initial stage of the pandemic, incorrectly suggesting to infected test-takers that they were not infected [39]. Even more importantly, a virus test only provides a momentary status, the individual can have the disease in the next few days after the test. Furthermore, practical difficulties in using the swab correctly might further increase the risk of underestimating virus infections. Currently available rapid antibody tests do not have the low rate of false positives that would be required for immunity guarantees, and they might therefore lure people into believing that they are immune when they are not. The seriousness of this problem in an early phase of the Covid-19 pandemic was well described by Ambati et al. [1]. With one test approved by the FDA in April 2020, 4% of the people who had not had the infection would incorrectly receive an immunity passport. If 2% of a population have had the infection, then two thirds of the passports will be issued to people who did not have the antibodies. (And, we may add, if 25% have had the infection, then about one in ten of the passports will be carried by a person without the antibodies.) Since then, antibody tests have been improved, and consequently the risk of issuing immunity passports to non-immune persons has been reduced. Nevertheless, both scientific and ethical difficulties remain. The comparative assessment of immunity acquired from the disease and from vaccination is far from trivial. Potential inequities in access to immunity passports can be considerable, and should be an important concern. [35, 48, 49]

All this adds up to make it essential that tested persons receive adequate information about how their test outcome should be interpreted, including help with applying this information to their own situation in life and—for the virus test—referral to adequate care. In general, self-testing not affiliated with a competent health-care provider who can offer such help should be avoided, in particular for serious diseases, since its use might otherwise lead to increased risk behaviour [54]. In situations where mass testing is needed, compromises may be necessary, but it should never be seen as a satisfactory or “normal” situation to provide testing for a serious disease disconnected from competent medical advice and access to treatment.

However, healthcare is nowhere apportioned solely according to needs. Its distribution depends on a complex combination of factors that shift with economic and political conditions as well as cultural habits and traditions. In almost all countries, a consumer demand for testing can be met not only by healthcare providers and public authorities, but also by businesses selling tests directly to consumers, usually without offering other medical services. It is clarifying to compare these activities with the sale of direct-to-consumer genetic tests for common multifactorial diseases such as coronary heart disease, stroke and arthritis. A person who receives test results showing that she has a lower than average risk of coronary heart disease will act most unwisely if she takes this as a pretext for adopting or continuing an unhealthy life-style. For instance, if she smokes, then she may very well run a considerably higher risk of cardiovascular disease than a non-smoker with a higher genetic predisposition. In fact, the general life-style recommendations to avoid cardiovascular disease are essentially the same for all, independently of genetic risk factors [9, 41]. There is a similar risk of over-interpretation of direct-to-consumer tests for a pandemic disease. A person who received a negative virus test or a positive antibody test may believe herself to be exempt from precautions that would have been needed to avoid contracting and spreading the disease.

In an initial phase of a pandemic, when resources for medically indicated testing is limited by lack of testing equipment, measures that ensure their use for the prioritized purposes are appropriate. Such measures would temporarily close down the direct-to-consumer market. However, when such drastic measures are no longer needed, it would seem more adequate to regulate the sale of self-testing equipment, imposing requirements of test quality, adequate information, and access to advice by a healthcare professional who is licensed in the customer’s country of residence.

## Testing of people with unclear legal status

Unauthorized immigrants are in a precarious situation with respect to healthcare. Many of them are in greater need of medical attention than other residents. For instance, migrants in Europe are disproportionately affected by tuberculosis, HIV, and hepatitis B and C [56, 63]. Their access to healthcare is limited or non-existent in many countries. This has negative health consequences not only for the adults who have chosen to migrate but also for their children. One example of this is lack of screening of pregnant migrants for diseases such as HIV and syphilis with significant mother-to-child transmission [61].

Many unauthorized immigrants have first arrived legally, but they have been denied asylum or overstayed a visa. In many European countries, newly arrived asylum seekers are routinely offered screening for a small number of infectious diseases, most often for tuberculosis [6, 33, 56]. Although migrants often appreciate this offer, they tend to prefer a general health check-up, which is of course also much more in line with their medical needs [6, 55]. Furthermore, participation in screening programs is often negatively affected by worries that positive test outcomes may reduce their chances of asylum. In general, unauthorized migrants tend to avoid seeking medical care, even if it is legally available, since they fear co-operation between healthcare and immigration law enforcement [26, 56]. Notably, this also affects children in these families.

Like everyone else, unauthorized residents are in need of medical attention in a pandemic. Testing and potential treatment of an infectious disease differs from most other types of healthcare in one important respect: it is obviously in the interest of society at large that unauthorized residents have access to these particular forms of healthcare. This should not be exaggerated. The common xenophobic scare of foreigners as the main source of dangerous infections is groundless. We are most likely to acquire disease agents from people with whom we have close physical contact or whom we meet on workplaces, in religious or recreational contexts, or in social life [4], p. 281. For instance, in countries where the pandemic is well controlled, screening (and/or quarantining) persons arriving from abroad can be an efficient means to prevent spread of the disease. Such screening should make no exception for asylum-seekers, but neither should it be targeted specifically at them. Returning citizens usually have a contact pattern that poses a greater risk of spreading the disease than the contact pattern of entrants with no close relations in the country. Nevertheless, inability to reach asylum-seekers, unauthorized immigrants, or any other group of residents can be a serious problem for the prevention of an infectious disease that potentially affects the whole population.

The experience with previous screening efforts strongly indicates that it will be difficult to reach unauthorized immigrants with a program that has virus testing as its sole medical offer. It would also seem difficult, from an ethical point of view, to justify offering them only such medical service that obviously also serves the self-interest of other residents [6]. A society that offers comprehensive medical services to unauthorized residents is in a much better position to reach them in a future pandemic than a society in which they have had little or no prior contact with healthcare providers.

In order to ensure that medical services, including testing in a pandemic, have the largest possible outreach among unauthorized residents, it is necessary to convincingly guarantee them that they do not risk legal sanctions by seeking healthcare for themselves or their children. Such a guarantee is also needed to ensure their participation in contact tracing. This is the firewall proposed by Joseph Carens:

“Democratic states can and should build a firewall between the enforcement of immigration law, on the one hand, and the protection of general human rights, on the other. We ought to establish as a firm legal principle that no information gathered by those responsible for protecting general human rights can be used for immigration enforcement purposes... If [irregular immigrants] need emergency health care, they should be able to seek help without worrying that the hospital will disclose their identity to those responsible for enforcing immigration laws.” [11], p. 133)

Protective measures, diagnosis, and treatment against a pandemic should obviously be on the safe side of this firewall from immigration law enforcement. The same applies to coercive public health restrictions after a positive test, which have to be clearly separated from legal sanctions connected with the person’s migration status. Maintaining the firewall between health measures and immigration law is in the interest of all residents in the countries where the unauthorized migrants live.

## Obligatory testing?

In a pandemic, extensive virus testing may be justified in order to identify and quarantine infected persons, either among the population at large or in certain specifically affected groups. In most cases, such testing can be performed voluntarily. However, a conflict can arise between an authority wanting to test the whole population, or all members of some subgroup of the population, and individuals who refuse to be tested, for some reason or other. Such refusals have been seen during the Covid-19 pandemic [36, 53]. They seem to be connected with the dissemination of conspiracy theories and pseudoscience relating to Covid-19 that has also given rise to organized resistance against preventive measures such as social distancing, face masks, and vaccination [22].

### Experience from other cases

It has in general been accepted that testing for a disease should be voluntary, and subject to informed consent. (For instance, genetic testing without informed consent has met with considerable resistance and criticism, see [7].) This applies not only to tests in a clinical setting but also to screening tests offered to segments of the general population [32, 44], p. 28. A Uruguayan decision in 2006 that made biennial breast cancer screening mandatory for female workers to get a “health card”, which all workers need, was challenged as unethical and ultimately defeated [2, 58].

During the Covid-19 pandemic, participation in screening programs appears to have been an offer that citizens in affected areas could not resist. Since testing for the virus is an essential part of a strategy to protect also others than the tested person, a case for obligatory screening can be made that is much stronger than for non-infectious diseases. This is not the first time that such a situation has arisen. Before discussing obligatoriness in the pandemic case we will therefore consider three other cases in which exceptions have been made to the general principle that medical measures should be voluntary: screening for other infectious diseases, vaccination, and treatment of infectious diseases. These three exceptions all have in common that they concern the health of other persons than those who are subject to the mandatory measures, and they can therefore provide interesting insights for the issue of testing in a pandemic.

*Screening for other infectious diseases*: Already in the 1930s and 1940s, some American states required couples wishing to marry to be screened for syphilis. After World War II, when many soldiers returned home with the disease, most states introduced such programs. However, these testing programs were shown to be inefficient due to the high costs and the small number of previously undiagnosed cases that were discovered. Similar programmes for pre-marital HIV screening were introduced in the 1980s, but they were also shown to be inefficient. Obligatory premarital test programmes have therefore gradually been terminated. In 2019, the last state (Montana) abolished its mandatory premarital blood tests [15, 17, 27, 43, 59].

In South Korea, massive mandatory HIV screening was conducted in the 1990s in large segments of the population that had been identified as risk groups. This included sailors on international routes and workers in food factories, hotels, and inns. In 1996, 4.9 million persons were tested. Partly because of human rights issues, mandatory screening was drastically reduced, beginning in 2000, and funding was reallocated to treatment and public health education. A study performed ten years later showed that the proportion of late presenters among patients who received an AIDS diagnosis increased after the extent of mandatory screening was reduced, suggesting that mandatory testing may in this case have been conducive to early diagnosis of the disease [13].

*Vaccination*: Already in the nineteenth century, scepticism against vaccination threatened the efficacy of vaccination campaigns, which led many countries to make vaccination obligatory. In the US, a landmark Supreme Court case in 1905, Jacobsen v. Massachusetts, upheld mandatory smallpox vaccination. Mandatory vaccination programs are still common in many parts of the world [19, 20]. They are supported by two major arguments [38, 50]. One of them is a paternalist argument, which applies to the vaccination of children. According to that argument, government should protect the health of children, and it can therefore override parental decisions that are detrimental to a child’s vital interests. The other argument is the disease transmission argument. According to that argument, everyone who can be vaccinated without significant health risks to themselves has a duty to be vaccinated in order not to contribute to the transmission of the disease. The role of the government is then to enforce that obligation. The disease transmission argument is particularly persuasive if the vaccine has less than 100% efficacy (so that others cannot fully protect themselves by being vaccinated) or if there are people who cannot, for medical reasons, be vaccinated.

Importantly, both the paternalist argument and the disease transmission argument are basically arguments for *universal* vaccination (with only medical exemptions). Both of them support mandatory vaccination only to the extent that obligatoriness is the best way to come as close as possible to universal vaccination. Whether or not that is the case is an empirical question.

In practice, mandatory vaccination usually comes with non-medical exemptions, i.e. exemptions that can be applicable to persons who are medically eligible to be vaccinated. In particular, such exemptions have been granted to persons who plead religious or ideological conviction as a reason not to be vaccinated [38]. Unfortunately, even a small proportion of these exemptions can create public health problems, since people with such convictions tend to cluster in religious congregations or other social networks within which disease outbreaks can occur [45]. There are indications that the introduction of a legal requirement in a programme that already offers vaccination to all children leads to an increased rate of vaccination [14]. However, the effects of sanctions on the affected children should also be taken into account. For instance, the common way to enforce vaccination requirements in the United States is to deny non-vaccinated children school admission [19]. This means that children’s right to education is withheld in response to their parents’ disobedience of the law, which is an obviously questionable practice.

Discussions on obligatory vaccination have mostly been focused on childhood vaccines, but there is one case of vaccination of adults that offers interesting analogies for Covid-19 testing (and also for Covid-19 vaccination). That case is vaccination against influenza. Influenza is mostly trivial for younger persons, but for the elderly it is a serious and often mortal infection. The immune systems of elderly people react poorly to vaccination, with protection rates often around 50–70%. Along with other measures such as improved hygiene, vaccination of health-care personnel is considered to be an efficient means to reduce risks for the elderly. Proposals have been made to make such vaccination a requirement for working in direct contact with vulnerable elderly patients [60].

*Treatment of infectious diseases:* Most countries have a legislation that can make testing and, if needed, subsequent treatment and/or quarantine obligatory for persons who can reasonably be expected to carry an infectious disease. This is based on a special feature of most infectious diseases, namely that the diseased person is not only a victim to the disease but also a vector who can spread it. (Tetanus, which is not transmitted via the common person-to-person routes, is a major exception.) This means that standard medical ethics with its exclusive focus on the individual patient and its emphasis on patient autonomy will have to be supplemented with measures aimed at protecting other potential victims [4, 5, 57].

Many, probably most, jurisdictions have regulations that empower medical authorities to compel persons with contagious diseases to be treated and/or quarantined. It should be noted that these regulations are typically applied in situations with a stronger reason to believe that the person is infected than in most screening situations. For instance, persons who had sexual intercourse with someone who carries a sexually transmitted disease often run a considerable risk of being infected.

Some jurisdictions mandate forced quarantine but not forced treatment. A patient can refuse treatment, but may then (as a last resort) be quarantined in order not to spread the disease to others. Forced quarantines have medieval roots, and are still practiced all around the world. In 1993 the New York commissioner of health was authorized to detain persons with tuberculosis for treatment in an isolation room on a guarded ward. Some of these persons were detained after they ceased being infectious in order to make sure that they completed their therapy, which is important to impede the development of multi-resistant bacteria [10, 21]. However, programs that have a strong focus on forced treatment or quarantine have been criticized for unnecessarily violating autonomy and also for discriminatory and stigmatizing practices. Although the need for such options as last resorts is generally recognized, some authors have warned against giving them a too prominent place on the agenda. For instance, some legislations have statutes specifically criminalizing behaviour that risks spreading AIDS, although such reckless behaviour has a relatively small role in disease transmission, and is already covered by existing criminal laws. Notably, such disease-specific laws tend to be directed at persons with an already stigmatized infection, such as AIDS. Concerns have also been raised that a focus on coercive measures can discourage people from contact with healthcare and, more generally, offset the trust necessary in disease prevention [19].

### Conclusions for the on-going pandemic

At least four important conclusions can be drawn from the comparative cases discussed in the previous section. First, it is important to distinguish between arguments for universal coverage of a diagnostic or therapeutic measure and arguments for making that measure obligatory. There are often good arguments why everyone, or everyone in a particular group, should undergo a particular medical procedure. This, however, is usually not a reason to make that procedure mandatory and legally enforced (for the group in question). In order to take the step from “desirable for everyone” to “legally required for everyone”, additional arguments are needed. Importantly, this applies even in cases when the measure has positive effects for others than those to whom it is applied, as is often the case for the diagnosis of serious infectious diseases. Even if it is a moral duty for instance to be tested, vaccinated, treated or quarantined for an infectious disease, that moral duty does not immediately translate into a justification of government enforcement of these measures. At the very least, *for obligatoriness of testing to be justified, it must lead to a demonstrably improved health outcome that could not be obtained with alternative, voluntary measures*.

Secondly*, coercive measures can contribute to stigmatization and discrimination*, which is a social ill that can hurt individuals as severely as disease. The risk of such effects is especially large for infections that particularly affect already discriminated groups, such as groups with an increased risk of AIDS. The surge in racism and violent hate crimes directed against Asian Americans during the Covid-19 pandemic shows the dire consequences of fuelling xenophobic myths during a pandemic [12, 25, 62]. Public health measures should protect the whole population and actively reject discriminatory actions or messages directed against any part of the population.

Thirdly, *when considering mandatory measures, possible evasive behaviours that may undermine them must be taken into account*. If healthcare is associated with coercive measures, people who fear those measures may shun healthcare. This can have serious consequences for them and their families and, if they are carriers of an infectious disease, also for others.

Fourth and finally, it is *imperative not to undermine public trust in healthcare and public health agencies*. Health work depends crucially on trust, and short-term gains for instance in the diagnosis of an infection or vaccination against it may turn out to be Pyrrhic victories if they are obtained at the price of decreased public trust.

Based on these considerations we are now going to consider three groups that have been, or can potentially become, subject to obligatory testing for SARS-CoV-2: residents in an area, employees on a workplace, and border crossers.

*Residents in an area*: Massive testing of residents in affected areas has been an important part of the response to the disease in China and some other Asian countries. The extent to which these screenings have been mandatory is not clear to us, but obviously the question arises whether they could justifiably be made so. No doubt, it can be highly desirable that all residents in an area are tested in order to ensure that quarantining and other protective measures are adequate and efficient. However, as we have already mentioned, it does not necessarily follow that the most efficient strategy is to force testing upon persons who for some reason refuse it. One important reason for this is that their response to enforced testing might very well be to escape to some area where testing is not imminent. From the viewpoint of disease containment, this could be a worse outcome than if they stay, untested, in their area of residence. There may also be a risk that legally enforced testing exacerbates stigmatization of residents in highly affected areas.

*Employees on a workplace*: Workplaces are among the most dangerous places for transmission of infections, which is the reason for the massive lockdowns and transitions to home working during the Covid-19 pandemic. On workplaces that are still running, measures to prevent spreading of the disease are usually needed. Such measures include strict policies for staying home with symptoms, increased opportunities to maintain proper distance, hygiene opportunities and routines, safety clothing, and if possible access to testing and health care services. Mandatory testing may in some cases be warranted, but it has to be used with caution. In workplaces as well as other settings, the advantages of mandatory testing for disease containment have to be weighed against its potential disadvantages in terms of privacy and autonomy, and also against the risk that negative test results give rise to a false sense of assurance, leading perhaps to more risky behaviour or less attention to symptoms among tested personnel. If the sanction against employees for refusing tests includes reduced salary, then the test must be considered mandatory.

In some workplaces the need for procedures to exclude infectious employees from the workplace is particularly large, making it justified for employers to require testing. For instance, this is so in healthcare and at other workplaces with close contacts with vulnerable persons, e.g. in elderly care. There is already considerable experience from mandatory vaccination of health care workers, for instance against influenza [60].

*Border crossers*: Screening of immigrants against communicable disease has a long history. Beginning in 1891, the American Public Health Service performed medical inspections on immigrants, with the explicit purpose to exclude potential entrants with a serious infectious disease such as tuberculosis, a venereal disease, or some other condition that would make them “likely to become a public charge” [3]. Today, medical examinations at borders are routinely undertaken only in acute epidemics. During the Covid-19 pandemic, many countries have introduced virus tests and/or quarantines for people crossing their borders. The usefulness of these measures is doubtful when they are applied to travellers who travel between countries that are about equally affected by the disease. To the extent that testing of border-crossers is performed, it should be based on criteria that can be justified in terms of the purpose of the testing. A major example of a reasonable criterion is where the traveller has been in the period of time up to her arrival that corresponds to the incubation period of the disease. Examples of discriminatory and therefore also ethically problematic criteria are citizenship, social status, and resources to pay for an exemption.

## Conclusions

We have discussed four major aspects of the question who should be tested in a pandemic.

Concerning *priority-setting when test resources are scarce*, we propose that priority should be given to virus tests rather than antibody tests, and that distributive issues should be managed according to the basic criterion of health care needs. In the case of Covid-19, the application of this principle will support the prioritization of those with high mortality risk due to age and pre-existing conditions, even if the expected loss of life-years is taken into account. In addition to the distributive issues, the overall outcome of the measures against the pandemic also has to be taken into account. This can justify testing that contributes to effective contact tracing and disease containment, even in population groups with comparatively low Covid-19 mortality. In particular in low-income countries with scarce healthcare resources it is important to weigh the positive effects of Covid-19 testing not only against different types of measures to curb the pandemic but also against other healthcare measures such as child vaccination.

Concerning commercial *direct-to-consumer test services*, we argue that in a phase with lack of test equipment, test resources should be reserved for medically prioritized testing. This will typically exclude direct-to-consumer services that are not part of healthcare provision. In a situation without such scarcity, the sale of self-testing equipment and services will have to be allowed, but they should be regulated in order to ensure test quality, adequate information, and access to advice by a healthcare professional.

Concerning *testing of people with unclear legal status*, primarily unauthorized immigrants and their children, we emphasize that like everyone else, these residents need access to adequate medical attention in a pandemic, which may include testing. Obviously, inability to reach this or any other group of residents can be a serious problem for the prevention of a contagious disease that potentially affects the whole population. A society that offers comprehensive medical services to unauthorized residents is in a much better position to reach them in a future pandemic than one in which they have had little or no prior contact with healthcare providers.

Finally, concerning *obligatory testing*, we emphasize that there are often strong reasons for universal testing, for instance in a residential area or on a workplace. However, the best way to achieve universal testing may not be to make testing mandatory. For instance, a person who refuses to be tested when testing is mandatory in her residential area may decide to escape to some other area, and possibly spread the disease to that area. However, in healthcare and other workplaces with close contacts with vulnerable persons, mandatory testing may be an adequate and justifiable measure.

## Data Availability

Not applicable.

## Abbreviations

AIDS: Acquired immunodeficiency syndrome
Covid-19: Coronavirus disease 2019
FDA: Food and drug administration
HIV: Human immunodeficiency virus
PCR: Polymerase chain reaction
RDT: Rapid diagnostic test
RNA: Ribonucleic acid
RT-PCR: Real-time polymerase chain reaction
SARS-CoV-2: Severe acute respiratory syndrome coronavirus 2
US: United States (of America

## Footnotes

1 https://www.who.int/news-room/detail/22-05-2020-at-least-80-million-children-under-one-at-risk-of-diseases-such-as-diphtheria-measles-and-polio-as-covid-19-disrupts-routine-vaccination-efforts-warn-gavi-who-and-unicef. Accessed 26 September 2020.
